# Accumulated promoter methylation as a potential biomarker for esophageal cancer

**DOI:** 10.18632/oncotarget.13510

**Published:** 2016-11-22

**Authors:** Xianzhen Peng, Hengchuan Xue, Lingshuang Lü, Peiyi Shi, Jianping Wang, Jianming Wang

**Affiliations:** ^1^ Department of Epidemiology, School of Public Health, Nanjing Medical University, Nanjing, 211166, China; ^2^ Department of Public Health and Preventive Medicine, Kangda College of Nanjing Medical University, Lianyungang, 222000, China; ^3^ Department of Thoracic Surgery, People's Hospital of Yangzhong, Yangzhong, 212200, China; ^4^ Department of Social Medicine and Health Education, School of Public Health, Nanjing Medical University, Nanjing, 211166, China; ^5^ The Innovation Center for Social Risk Governance in Health, School of Public Health, Nanjing Medical University, Nanjing, 211166, China

**Keywords:** esophageal cancer, epigenetics, methylation, next-generation sequencing, diagnosis

## Abstract

We performed a two-stage molecular epidemiological study to explore DNA methylation profiles for potential biomarkers of esophageal squamous cell carcinoma (ESCC) in a Chinese population. Infinium Methylation 450K BeadChip was used to identify genes with differentially methylated CpG sites. Sixteen candidate genes were validated by sequencing 1160 CpG sites in their promoter regions using the Illumina MiSeq platform. When excluding sites with negative changes, 10 genes (*BNIP3, BRCA1, CCND1, CDKN2A, HTATIP2, ITGAV, NFKB1, PIK3R1, PRDM16* and *PTX3*) showed significantly different methylation levels among cancer lesions, remote normal-appearing tissues, and healthy controls. *PRDM16* had the highest diagnostic value with the AUC (95% CI) of 0.988 (0.965–1.000), followed by *PIK3R1*, with the AUC (95% CI) of 0.969 (0.928–1.000). In addition, the methylation status was higher in patients with advanced cancer stages. These results indicate that aberrant DNA methylation may be a potential biomarker for the diagnosis of ESCC.

## INTRODUCTION

Esophageal cancer is one of the most common cancers worldwide, with approximately 456,000 new cases and 400,000 deaths in 2012 [1, 2]. Esophageal squamous cell carcinoma (ESCC) is the most prevalent esophageal cancer in the world, especially in Asian countries [3, 4]. ESCC is highly invasive and rapidly metastatic, often resulting in a poor postoperative quality of life [5, 6]. In spite of clinical advances in the field of oncology, the overall long-term survival rates of ESCC remain dismal [7]. If patients were diagnosed and treated at an early stage, the five-year survival rate after endoscopic mucosectomy could reach 100% [8]. Therefore, there is an urgent need to identify sensitive and specific biomarkers for the early diagnosis of ESCC.

One of the early events that occur during carcinogenesis are the epigenetic changes [9, 10]. Epigenetic modifications cause heritable changes to cells without changes to DNA sequence. Epigenetic modifications, such as methylation, histone modifications, DNA replication timing, nucleosome positioning, or heterochromatization, result in selective gene expression or repression [9, 11]. DNA methylation is one of the most extensively characterized epigenetic modifications [12, 13]. Aberrant DNA methylation has been associated with various human diseases, including cancer [14], autoimmune diseases [15], mental illness [16], and cardiovascular diseases [17]. Large-scale methylation analysis of human genomic DNA may provide a better understanding of the molecular mechanisms involved in the esophageal carcinogenesis [18].

In this epidemiological study, we analyzed the impact of aberrant DNA methylation levels on the clinical and pathological features of ESCC in a Chinese population, and we investigated the methylation profile as a potential biomarker for the diagnosis of esophageal cancer.

## RESULTS

### Identification of candidate genes

The heat map of hierarchical clustering of methylation according to the data from the Infinium Methylation 450K array is shown in Supplementary Figure 1. Based on diffScore, delta β and gene function, we selected 16 candidate genes (*RASSF1, PIK3R1, ITGAV, NFKB1, TAP2, APC, BRCA1, CCND1, CDH1, CDKN2A, BNIP3, HTATIP2, PRDM16, PTEN, PTX3* and *SOCS1*) for validation (Supplementary Figure S2).

### Validation of methylated CpG sites

**Figure 1:**
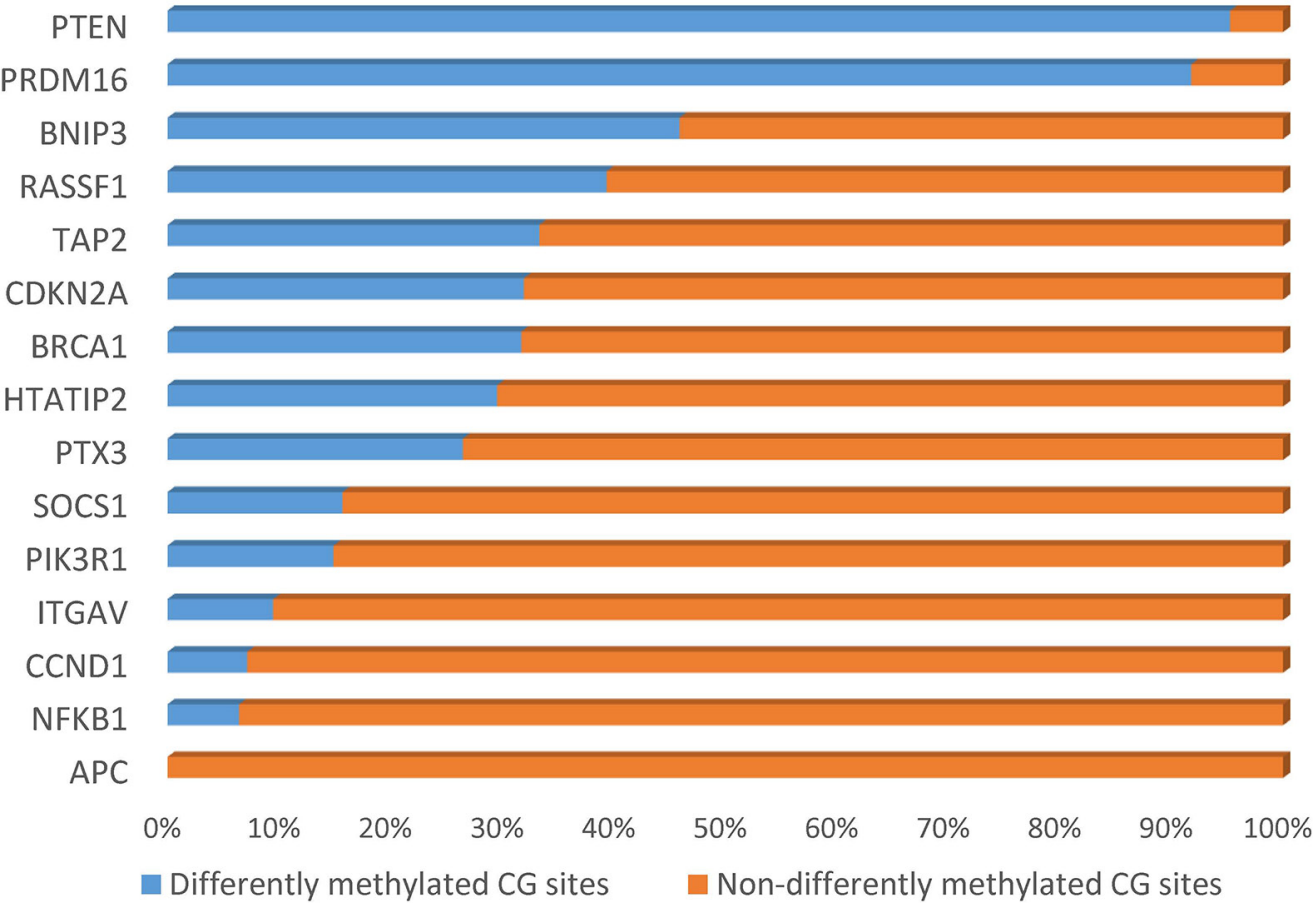
Percent of differentially methylated sites in each candidate gene

**Figure 2:**
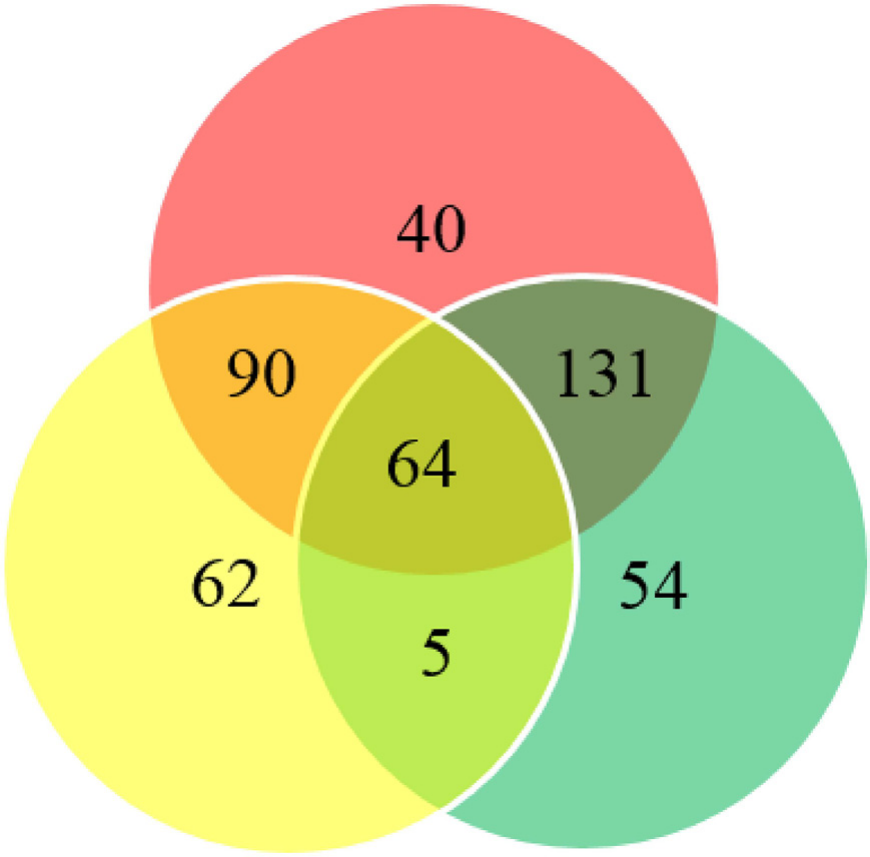
Venn diagram summarizing the differentially methylated sites Red circle indicates differentially methylated sites between cancer and healthy control tissues; yellow circle indicates differentially methylated sites between cancer and remote normal-appearing tissue; green circle indicates differentially methylated sites between remote normal-appearing and healthy control tissues.

We collected 43 cancer lesion samples, 43 remote normal-appearing esophageal tissues, and 10 healthy control tissues. The patients included 28 males and 15 females, with the age ranging from 46 to 81 years (Table 1). We also recruited 10 healthy controls, including 7 males and 3 females, with the age ranging from 42 to 74 years (mean ± standard deviation: 58.8 ± 9.2 years). We sequenced 1160 CpG sites in the promoter region of 16 candidate genes. After excluding loci with low calling rate, 961 CpG sites in 15 genes met the requirements for further analysis (Table 2). There were 33.82% (325/961) CpG sites showing significant differences in the distribution of methylation between ESCC and normal esophageal tissues (*P <* 0.05). The proportion of differentially methylated sites in each gene is shown in Figure 1. There were 195 sites having 2 to 10 fold changes and 58 sites having more than 10 fold changes between ESCC and normal esophageal tissues. 299 out of differentially methylated 325 CpG sites (92 %) had higher methylation level in ESCC samples compared with healthy controls. In addition, 254 CpG sites had significantly different methylation between remote normal-appearing tissues and health controls, and 221 CpG sites had significantly different methylation status between ESCC and remote normal-appearing tissues. The above results are summarized in a Venn diagram in Figure 2. There were 64 CpG sites differentially methylated between these three groups (cancer lesions, remote normal-appearing samples, and health controls). Among them, 54 CpG sites were located in the gene of *PRDM16*.

**Table 1:** Clinical characteristics of patients

No.	Gender	Age	Tumor location	Smoking	Drinking	TNM	G stage (histologic grade)
1	Female	78	Lower	No	No	T2N0M0	G3
2	Female	68	Lower	No	No	T1N0M0	G2
3	Male	72	Middle	Yes	No	T1N0M0	G2
4	Male	64	Middle	Yes	Yes	T3N1M0	G2
5	Male	69	Middle	No	No	T2N0M0	G2
6	Male	62	Middle	No	No	T3N0M0	G2
7	Male	57	Middle	Yes	Yes	T4N0M0	G2
8	Female	58	Upper	No	No	T2N1M0	G2
9	Female	73	Upper	No	No	T3N0M0	G2
10	Female	68	Middle	No	No	T3N1M0	G2
11	Female	68	Upper	No	No	T3N1M0	G2
12	Female	69	Middle	No	No	T2N1M0	G2
13	Female	64	Middle	No	No	T1N0M0	G2
14	Male	64	Lower	Yes	Yes	T2N2M0	G2
15	Female	61	Lower	No	No	T3N1M0	G3
16	Male	75	Middle	Yes	No	T3N1M0	G3
17	Male	54	Middle	Yes	No	T3N0M0	G1
18	Female	65	Middle	No	No	T2N0M0	G2
19	Male	54	Lower	Yes	Yes	T3N1M0	G2
20	Male	62	Middle	No	No	T3N1M0	G3
21	Male	78	Middle	Yes	No	T3N1M0	G2
22	Male	63	Middle	Yes	No	T2N0M0	G3
23	Female	76	Upper	Yes	Yes	T1N0M0	G2
24	Male	59	Middle	Yes	Yes	T2N1M0	G3
25	Female	60	Middle	No	No	T2N1M0	G2
26	Male	67	Middle	No	No	T2N0M0	G3
27	Male	60	Middle	No	No	T2N1M0	G3
28	Male	60	Middle	No	No	T3N1M0	G3
29	Male	67	Middle	Yes	Yes	T3N0M0	G2
30	Male	46	Middle	Yes	Yes	T3N1M0	G2
31	Male	81	Middle	No	Yes	T2N0M0	G2
32	Male	61	Middle	No	Yes	T1N1M0	G3
33	Male	46	Middle	No	No	T3N1M0	G3
34	Male	65	Middle	Yes	Yes	T3N1M0	G2
35	Female	70	Middle	No	No	T3N1M0	G3
36	Male	67	Lower	No	No	T1N0M0	G3
37	Female	60	Upper	No	No	T1N0M0	G2
38	Male	64	Lower	No	Yes	T3N0M0	G2
39	Male	77	Lower	Yes	No	T3N1M0	G2
40	Male	74	Middle	Yes	Yes	T3N2M0	G3
41	Male	71	Lower	Yes	Yes	T3N3M0	G3
42	Female	71	Middle	No	No	T1N0M0	G2
43	Male	60	Middle	Yes	Yes	T2N1M1	G2

**Table 2:** Sequenced sites of selected genes

Genes	Fragment	Start/Stop	Size (bp)	Number of CpG sites
RASSF1	RASSF1_M1	50377767/50378028	261	21
	RASSF1_M2	50378005/50378218	213	23
	RASSF1_M3	50378194/50378472	278	27
	RASSF1_M5	50375039/50375295	256	23
	RASSF1_M6	50374899/50375126	227	19
	RASSF1_M7	50374706/50374925	219	29
	RASSF1_M9	50374301/50374516	215	13
PIK3R1	PIK3R1_M2	67511168/67511412	244	22
	PIK3R1_M4	67511596/67511806	210	23
	PIK3R1_M5	67511286/67511520	234	21
	PIK3R1_M6	67511047/67511305	258	22
	PIK3R1_M7	67512226/67512438	212	18
	PIK3R1_M8	67584255/67584471	216	15
ITGAV	ITGAV_M1	187454700/187454960	260	21
	ITGAV_M2	187454936/187455177	241	32
	ITGAV_M3	187455157/187455369	212	21
NFKB1	NFKB1_M2	103422534/103422795	261	37
	NFKB1_M3	103422775/103422981	206	23
	NFKB1_M4	103423077/103423302	225	18
TAP2	TAP2_M1	32806418/32806681	263	12
APC	APC_M2	112073375/112073585	210	15
BRCA1	BRCA1_M1	41275281/41275523	242	18
	BRCA1_M3	41275011/41275281	270	11
	BRCA1_M4	41275268/41275528	260	12
CCND1	CCND1_M3	69458670/69458890	220	14
CDKN2A	CDKN2A-2	21993123/21993331	208	20
	CDKN2A-4	21993770/21993957	187	16
	CDKN2A-6	21994239/21994504	265	26
	CDKN2A-7	21994477/21994700	223	11
	CDKN2A_M8	21972954/21973198	244	11
	CDKN2A_M9	21974670/21974872	202	15
	CDKN2A_M10	21974852/21975095	243	20
BNIP3	BNIP3-1	133795927/133796159	232	10
	BNIP3-3	133796371/133796631	260	33
	BNIP3-6	133797020/133797250	230	26
	BNIP3-7	133797230/133797402	172	16
HTATIP2	HTATIP2-1	20385087/20385355	268	24
	HTATIP2-2	20385336/20385546	210	20
PRDM16	PRDM16-1	2983847/2984081	234	14
	PRDM16-5	2984736/2984979	243	29
	PRDM16-7	2985182/2985386	204	19
	PRDM16-8	2985367/2985573	206	16
	PRDM16-9	2985553/2985775	222	19
PTEN	PTEN-1	89623758/89624026	268	21
PTX3	PTX3-1	157155257/157155524	267	9
	PTX3-2	157155500/157155711	211	25
SOCS1	SOCS1-1	11349069/11349310	241	20
	SOCS1-3	11349540/11349759	219	31

### Diagnostic value analysis

We further analyzed the cumulative methylation levels by considering multiple CpG sites in each gene. The diagnostic values of selected CpG sites and genes were estimated based on three different models.

### Model 1

We calculated the cumulative methylation by summarizing the frequency of all CpG sites in each gene. Nine genes (*APC, BNIP3, BRCA1, CCND1, CDKN2A, HTATIP2, ITGAV, PRDM16* and *TAP2*) showed significantly different cumulative methylation levels among the three groups. The methylation levels of *APC, ITGAV, PRDM16* and *PTX3* were significantly different between esophageal cancer and healthy control tissues (Table 3). The AUC (95% CI) of each gene in the diagnosis of ESCC is listed in Table 4. The *PRDM16* gene showed the highest diagnostic value with the AUC (95% CI) of 0.958 (0.906–1.000), followed by *ITGAV*, with the AUC (95% CI) of 0.779 (0.651–0.907).

**Table 3:** Comparison of cumulative methylation levels of multiple CpG sites in each gene using different models

Gene	Model A								Model B								Model C							
	Cumulative methylation level			F	*P*	*P*			Cumulative methylation level			F	*P*	*P*			Cumulative methylation level			F	*P*	*P*		
	*N* (*n*=10)	A (*n*=43)	T (*n*=43)			*N vs.* A	A *vs.* T	*N vs.* T	*N* (*n*=10)	A (*n*=43)	T (*n*=43)			*N vs.* A	A *vs.* T	*N vs.* T	N (*n*=10)	A (*n*=43)	T (*n*=43)			*N vs.* A	A *vs.* T	*N vs.* T
APC	0.108	0.118	0.199	6.739	0.034	0.355	0.405	0.04	-	-	-	-	-	-	-	-	-	-	-	-	-	-	-	-
BNIP3	6.902	8.833	8.041	9.57	0.008	0.045	0.031	1	1.251	2.505	2.579	18.411	<0.001	<0.001	1	<0.001	1.251	2.505	2.579	18.411	<0.001	<0.001	1	<0.001
BRCA1	22.537	22.669	20.809	14.314	0.001	1	<0.001	0.475	10.075	9.99	9.038	32.385	<0.001	1	<0.001	0.001	0.88	0.898	0.896	6.655	0.036	0.03	1	0.114
CCND1	0.134	0.144	0.133	7.392	0.025	0.265	0.033	1	0.004	0.008	0.008	8.725	0.013	0.013	1	0.017	0.004	0.008	0.008	8.725	0.013	0.013	1	0.017
CDKN2A	5.913	6.941	7.23	15.116	0.001	0.055	0.001	1	1.718	2.183	2.662	10.386	0.006	0.102	0.009	1	0.352	0.905	1.68	20.527	<0.001	<0.001	1	<0.001
HTATIP2	0.363	0.525	0.527	8.63	0.013	0.01	1	0.052	0.082	0.155	0.17	16.578	<0.001	<0.001	1	<0.001	0.082	0.155	0.17	16.578	<0.001	<0.001	1	<0.001
ITGAV	0.614	0.715	0.722	10.101	0.006	0.005	1	0.013	0.029	0.059	0.065	14.9	0.001	0.003	1	<0.001	0.029	0.059	0.065	14.9	0.001	0.003	1	<0.001
NFKB1	0.687	0.777	0.942	1.731	0.421	-	-	-	0.007	0.042	0.058	14.347	0.001	0.001	1	0.001	0.007	0.042	0.058	14.347	0.001	0.001	1	0.001
PIK3R1	1.291	1.357	1.969	2.422	0.298	-	-	-	0.057	0.153	0.213	22.693	<0.001	<0.001	0.576	<0.001	0.041	0.142	0.204	25.481	<0.001	<0.001	0.376	<0.001
PRDM16	9.155	13.202	20.756	35.643	<0.001	0.003	0.001	<0.001	6.724	10.686	18.062	36.8949	<0.001	0.003	<0.001	<0.001	5.86	9.867	17.251	37.181	<0.001	0.002	<0.001	<0.001
PTEN	0.381	0.534	1.666	5.182	0.075	-	-	-	0.374	0.53	1.659	4.886	0.087	-	-	-	0.374	0.53	1.659	4.886	0.087	-	-	-
PTX3	0.468	1.053	1.649	0.947	0.623	-	-	-	0.301	0.222	0.391	6.239	0.044	0.125	1	0.037	0.03	0.222	0.391	6.239	0.044	0.125	1	0.037
RASSF1	2.535	3.482	8.962	3.598	0.165	-	-	-	1.804	2.71	6.898	2.871	0.238	-	-	-	1.803	2.71	6.898	2.871	0.238	-	-	-
SOCS1	1.328	2.321	2.391	3.888	0.143	-	-	-	0.085	0.276	0.316	5.436	0.066	-	-	-	0.084	0.276	2.338	5.436	0.066	-	-	-
TAP2	5.712	5.719	5.362	32.904	<0.001	1	<0.001	0.003	2.369	2.325	2.152	44.501	<0.001	0.301	<0.001	<0.001	-	-	-	-	-	-	-	-

N: healthy control tissues; A: remote normal-appearing tissues; T: cancer tissue

**Table 4:** Diagnostic values of selected genes for esophageal cancer using different models

Gene	No. of sample		Model A			Model B			Model C		
	Cases	Controls	AUC	95% CI	*P*	AUC	95% CI	*P*	AUC	95% CI	*P*
APC	43	10	0.73	0.535-0.925	0.024	–	–	–	–	–	–
BNIP3	43	10	0.556	0.388-0.723	0.585	0.856	0.749-0.962	0.001	0.876	0.775-0.978	< 0.001
BRCA1	43	10	0.326	0.180-0.471	0.088	0.13	0.033–0.228	< 0.001	0.712	0.529–0.895	0.039
CCND1	43	10	0.521	0.301–0.741	0.838	0.799	0.648–0.949	0.003	0.817	0.667–0.966	0.002
CDKN2A	43	10	0.437	0.290–0.585	0.539	0.451	0.301–0.601	0.633	0.912	0.832–0.992	< 0.001
HTATIP2	43	10	0.698	0.558–0.837	0.053	0.872	0.766–0.978	< 0.001	0.881	0.779–0.983	< 0.001
ITGAV	43	10	0.779	0.651–0.907	0.006	0.879	0.783–0.975	< 0.001	0.898	0.807–0.988	< 0.001
NFKB1	43	10	0.605	0.430–0.779	0.306	0.855	0.753–0.956	0.001	0.869	0.772–0.966	< 0.001
PIK3R1	43	10	0.635	0.464–0.806	0.187	0.93	0.863–0.998	< 0.001	0.969	0.928–1.000	< 0.001
PRDM16	43	10	0.958	0.906–1.000	< 0.001	0.967	0.921–1.000	< 0.001	0.988	0.965–1.000	< 0.001
PTEN	43	10	0.663	0.491–0.835	0.112	0.66	0.491–0.830	0.117	0.675	0.505–0.845	0.088
PTX3	43	10	0.593	0.430–0.756	0.363	0.737	0.604–0.871	0.02	0.749	0.617–0.880	0.015
RASSF1	43	10	0.681	0.528–0.835	0.076	0.66	0.488–0.833	0.117	0.676	0.502–0.850	0.086
SOCS1	43	10	0.628	0.472–0.784	0.211	0.74	0.600–0.897	0.019	0.752	0.615–0.890	0.014
TAP2	43	10	0.14	0.031–0.248	< 0.001	0.028	0.000–0.066	< 0.001	–	–	–

### Model 2

By excluding non-significantly differentiated CpG sites, we calculated the cumulative methylation by summarizing the frequency of significant CpG sites in each gene. Eleven genes (*BNIP3, BRCA1, CCND1, CDKN2A, HTATIP2, ITGAV, NFKB1, PIK3R1, PRDM16, PTX3* and *TAP2*) showed significant differences in methylation between groups. The number of differently methylated genes increased to 10 (*BNIP3, BRCA1, CCND1, HTATIP2, ITGAV, NFKB1, PIK3R1, PRDM16, PTX3* and *TAP2*) between ESCC and healthy control tissues (Table 3). The AUC values (95% CI) of each gene in the diagnosis of ESCC are listed in Table 4. Methylation of *PRDM16* gene had the highest diagnostic value, with the AUC (95% CI) of 0.967 (0.921–1.000), followed by *PIK3R1*, with the AUC (95% CI) of 0.930 (0.863–0.998).

### Model 3

**Figure 3:**
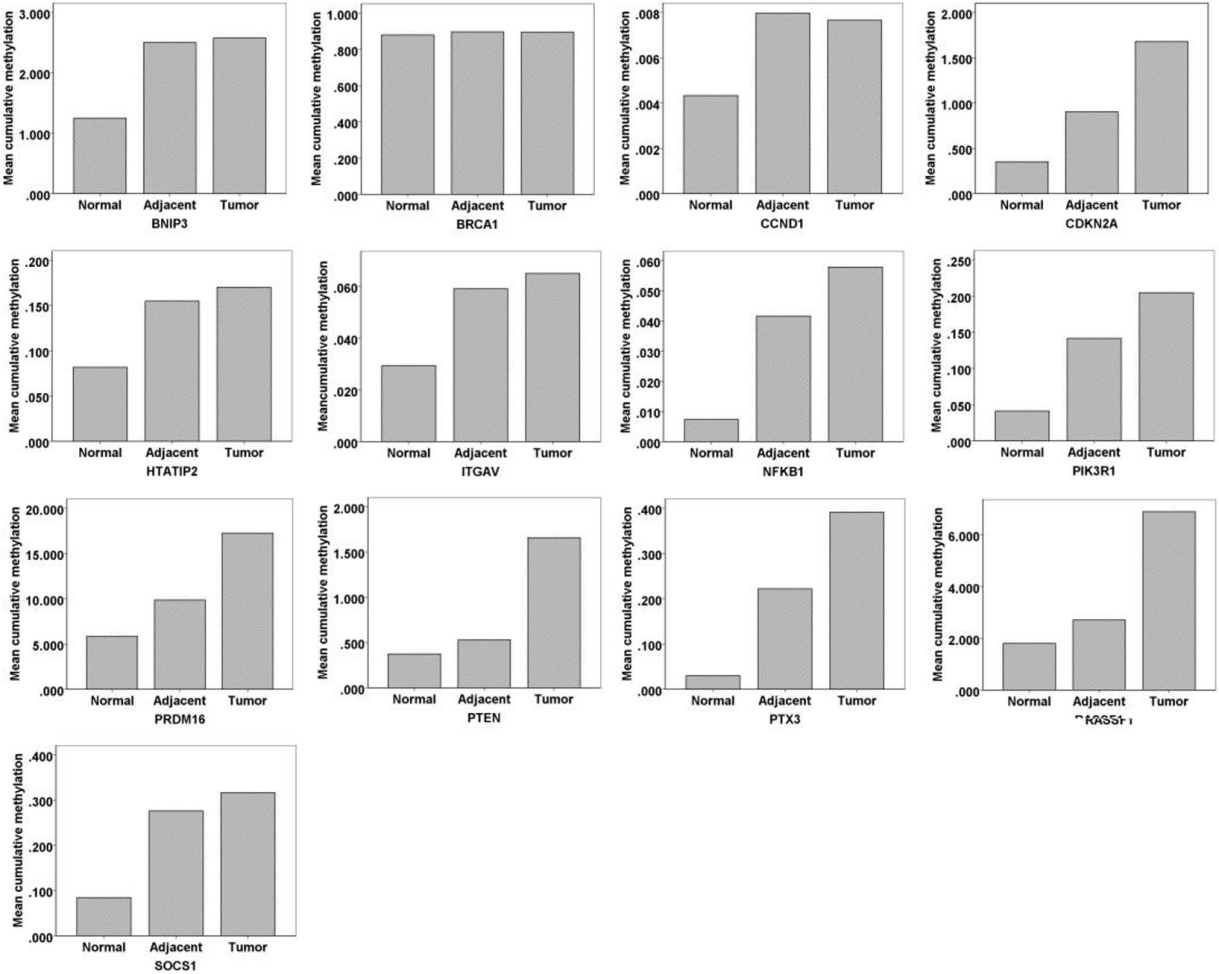
The cumulative methylation levels of multiple CpG sites in each gene

We further excluded CpG sites with negative correlations and kept 299 sites for analysis. Ten genes (*BNIP3, BRCA1, CCND1, CDKN2A, HTATIP2, ITGAV, NFKB1, PIK3R1, PRDM16* and *PTX3*) had significantly different methylation status among the three groups. The methylation levels of *BNIP3, CCND1, CDKN2A, HTATIP2, ITGAV, NFKB1, PIK3R1, PRDM16* and *PTX3* were significantly different between esophageal cancer and healthy control tissues (Table 4). The AUC (95% CI) of each gene in the diagnosis of ESCC is listed in Table 4. The methylation of PRDM16 gene showed the highest diagnostic value, with the AUC (95% CI) of 0.988 (0.965–1.000), followed by *PIK3R1*, with the AUC (95% CI) of 0.969 (0.928–1.000). Compared with findings using model 1 and model 2, the AUC of each gene in model 3 has greatly increased. Especially for *BRCA1* and *CDKN2A*, the AUC increased from less than 0.5 to 0.712 and 0.912, respectively. Based on the model 3, the cumulative methylation level of most genes increased with the histologic changes from normal to normal-appearing tissues and cancer lesions (Figure 3). To avoid false positives caused by multiple comparisons between groups, we used the Bonferroni correction method. Using Bonferroni correction, 49 CpG sites in 4 genes were significant, including 1 site in *BNIP3*, 1 site in *PIK3R1*, 46 sites in *PRDM16*, and 1 site in *SOCS1*. The cumulative methylation levels of *PRDM16* were significantly different among the three groups (F = 38.445, *P <* 0.001). The AUC of *PRDM16* was 0.963 (95% CI: 0.914–1.000).

### Methylation status and clinical characteristics

**Figure 4:**
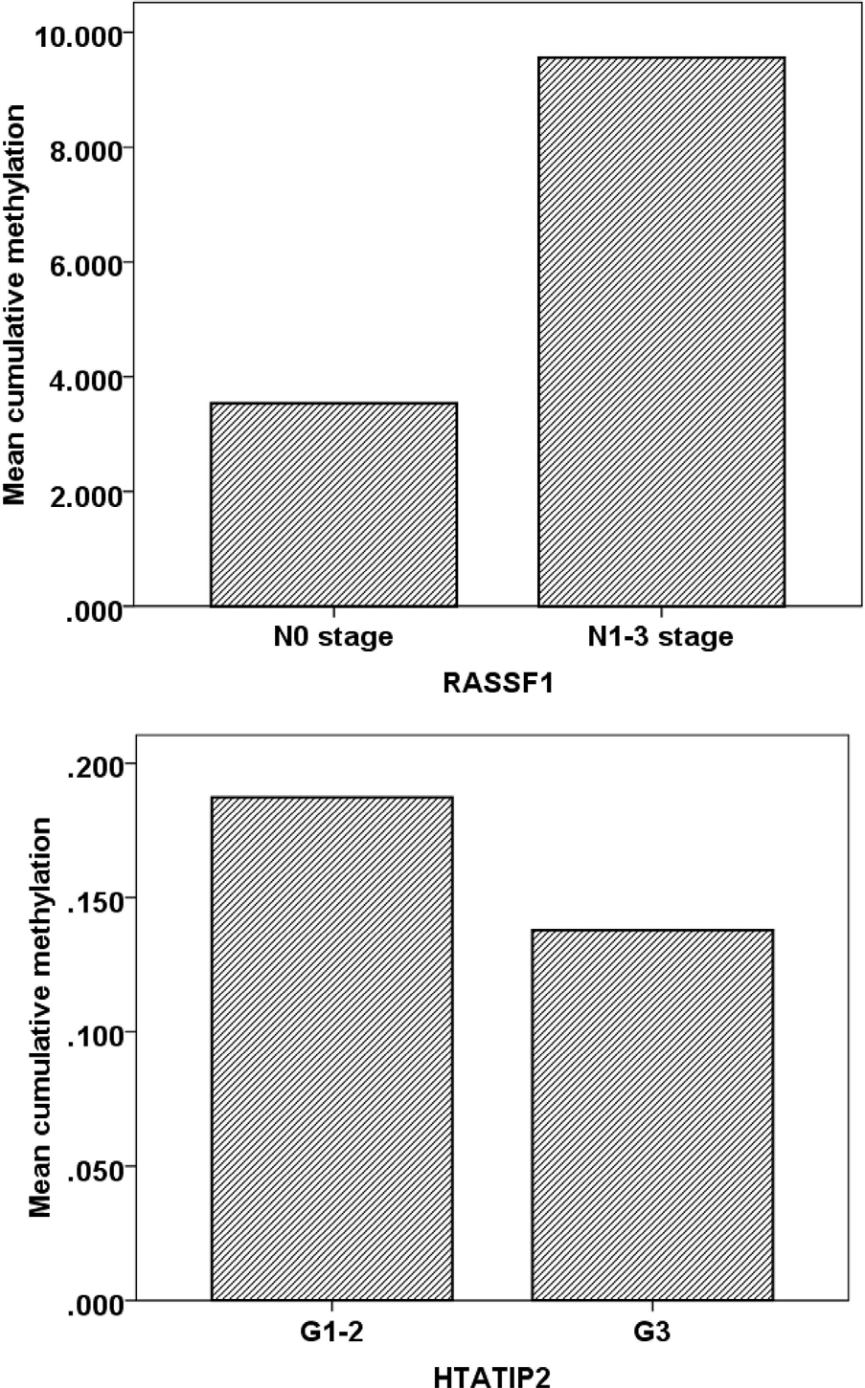
The relationship between clinical characteristics and DNA methylation in cancer lesions

The methylation frequency was higher in patients at advanced cancer stages. For example, samples from patients with N1-3 stage had an average cumulative methylation value of 9.56 in *RASSF1* gene, which was significantly higher than that in patients at N0 stage (cumulative methylation value: 3.54). For *HTATIP2* gene, samples from patients at G1-2 stages also had a significantly higher cumulative methylation level compared with patients at G3 stage (*P <* 0.05, Figure 4). The cumulative methylation levels of these genes did not correlate with patient's gender (male and female) and age (< 60 and >= 60 years).

### Protein expression and methylation status

Next, we analyzed protein levels of RASSF1, PIK3R1 and PTEN by immunohistochemistry. In the esophageal cancer lesions, PIK3R1 was expressed in 65.5% (19/29) cases (+: 18; ++: 1; +++: 0), PTEN was expressed in 48.3% (15/31) cases (+: 14; ++: 1; +++: 0), and RASSF1 was expressed in 56.7% (17/30) cases (+: 14; ++: 3; +++: 0). We observed a negative correlation between the methylation level and the IOD, but the coefficient was not significant (*RASSF1*: r = −0.122, *P* = 0.521; *PIK3R1*: r = −0.215, *P* = 0.264; *PTEN*: r = −0.095, *P* = 0.619). The methylation level of *RASSF1* gene was significantly higher in samples with negative expression than in samples with positive expression (*P* = 0.022).

## DISCUSSION

When cancer occurs, a massive global hypomethylation is frequently observed, while certain genes can be hypermethylated at the CpG islands [19]. Previous studies have indicated aberrant DNA methylation in esophageal cancer; however, those studies have focused on limited CpG sites [20–22]. In this study, we used a two-stage study design, sequenced 1160 CpG sites in the promoter region of 16 candidate genes, and demonstrated that aberrant DNA methylation can be a potential biomarker for esophageal cancer.

Compared with other methods, such as MSP, Q-PCR, MethyLight or bisulfite pyrosequencing, NGS used in this study can capture full sample diversity with small amounts of DNA. In addition, NGS can enhance epigenetic analyses with high coverage density and flexibility, which help advance our understanding of epigenetics at the genomic level [23]. A fluorescently labeled reversible terminator is utilized in this system, allowing for the accrual of qualitative and quantitative information of nucleic acid at an incredible throughput while incurring relatively limited costs [24].

One of the most robust epigenetic marks found in this study was *PRDM16* gene. *PRDM16* is located near the 1p36.3 breakpoint, encoding a zinc finger transcription factor and contains an N-terminal PR domain. It is known to be a fusion partner of *RPN1, RUNX1* and other genes in hematopoietic malignancies [25]. The malfunction of *PRDM16* is related to a poor prognosis of cancer patients [26]. For example, *PRDM16* is often methylated in lung cancer cells, with downregulated protein expression [27]. The demethylation drug 5-aza-2′-dC upregulates PRDM16 expression and suppresses growth of lung cancer cells [27].

Other genes with a higher AUC (over 0.9) for distinguishing ESCC were *PIK3R1* and *CDKN2A*. *PIK3R1* encodes a p85 regulatory subunit alpha and appears to play a tumor suppressor role because PI3K subunit p85α (p85α) regulates and stabilizes p110α [28]. A previous study has reported that the expression of *PRK3R1* negatively correlates with hypermethylation of CpG sites in *PIK3R1* [29]. Our results also showed similar negative correlations, although they were not statistically significant; this may be due to the limited sample size. CDKN2A blocks phosphorylation of the Rb protein and inhibits cell cycle progression. *CDKN2A* is aberrantly methylated in esophageal cancer [30], and is associated with metastatic and invasive phenotypes [31]. Similar *CDKN2A* methylation patterns have been observed in gastric and nasopharyngeal carcinoma [32, 33]. As the regional lymph node metastasis is associated with the patient's prognosis, the methylation status of these genes might be used to assess the possibility of recurrence and metastasis of ESCC patients and also help to implement proper medications. Moreover, our study shows that the methylation levels of selected genes, such as *RASSF1* and *HTATIP2,* change with the cancer stages, indicating their potential values in the prognosis of ESCC.

There are several limitations in this study. First, the bisulfite conversion efficiency is critical for the accuracy and the reliability of the results. The incomplete conversion of unmethylated cytosine to uracil or inappropriate conversion of methylcytosine to thymine can cause over- or underestimation of the methylation level. It is also noteworthy that the bisulfite conversion technique cannot be used to discriminate the methylated cytosine from 5-hydroxymethylcytosine [34]. Second, the false positives may be caused by multiple comparison when we compared various CpG sites between groups. We used the Bonferroni correction method to adjust for the test level; however, this is an overcorrection when the tests are correlated [35]. Third, aberrant DNA methylation usually occurs somatically in cancer cells and can also be detected in peripheral blood samples [36]. To evaluate the clinical use of aberrant DNA methylation, a blood-based assay is preferable, since it uses a far less invasive procedure.

In conclusion, aberrant DNA methylation is a promising biomarker that has a good predictive value for identifying esophageal cancer in a molecular diagnostic laboratory. The hypermethylation status of *PRDM16, PIK3R1,* and *CDKN2A* genes might be used as a potential biomarker for the diagnosis of ESCC.

## MATERIALS AND METHODS

### Study design

First, we used the Illumina Infinium 450K Methylation Beadchip to construct a genome-wide DNA methylation profile. Then, candidate genes were selected for the validation using the Next-Generation Sequencing (NGS) platform (Illumina MiSeq platform).

### Study subjects

This study was approved by the Ethics Committee of Nanjing Medical University. Written informed consent was obtained from all participants. The methods were carried out in accordance with the approved guidelines. Esophageal cancer patients were recruited in the Yangzhong People's Hospital from 2012 to 2016. Yangzhong is an area with high morbidity and mortality rates of the upper digestive tract cancers [37]. The inclusive criteria were: (1) Patients were diagnosed as ESCC with histopathological evidence; (2) All patients were of Chinese Han origin living in Yangzhong longer than five years; (3) Patients underwent esophagectomy and the lesions were eligible for sampling; (4) None of the patients had received preoperative radiotherapy or chemotherapy. Tissues in the center of the cancer lesion and remote normal-appearing esophagus were excised and immediately stored in -80°C freezer. Healthy control esophageal tissues were collected from individuals who had no cancer history and participated in a screening program for upper digestive tract cancers.

### DNA extraction

Genomic DNA was extracted from tissues using the QIAmp DNA Mini Kit (Qiagen, Hilden, Germany). The quality and concentration were evaluated with Thermo NanoDrop 2000-1 spectrophotometer (NanoDrop Technologies, Montchanin, DE, USA).

### Infinium methylation 450K array

We used the Infinium 450K Methylation Beadchip (Illumina, San Diego, CA, USA) to evaluate the methylation status of five paired tumor samples and corresponding remote normal-appearing esophagus tissues, along with two normal controls from the healthy population.

### Next-generation sequencing (NGS)

#### Primer design and optimization

Genomic regions were analyzed and transformed to bisulfite-converted sequences by gene CpG software. The primers were designed by the Gensky Bio-Tech Co., Ltd. (Shanghai) to amplify regions of interest from the bisulfite converted DNA. Different sets of primers were compared using 1 ng bisulfite modified positive and negative control DNA samples. The final optimized primers are listed in Table 5.

**Table 5:** Primers designed for multiplex PCR

Genes	Fragment	Forward primer	Reverse primer
RASSF1	RASSF1_M1	AAGGAGGGAAGGAAGGGTAAG	CCAACTCCCRCAACTCAATAAAC
	RASSF1_M2	GGGGAGTTTGAGTTTATTGAGTTG	CCCAAATAAAATCRCCACAAAAATC
	RASSF1_M3	GATTTTTGTGGYGATTTTATTTGG	TACATATAAACAACCACCTCTACTCATCT
	RASSF1_M5	GGTAAGYGTATAAGAGTGGTTTTTGGT	AACAAACCACAATACAAACATTCTC
	RASSF1_M6	GATTTAGTTTTTGTTTTATTGGGGTAG	ACCCAAACTAACCCAAACTCC
	RASSF1_M7	GTTGTTTTAGGTTATTTYGAAAGAAGG	CTACCCCAATAAAACAAAAACTAAATC
	RASSF1_M8	GTTAGGAGGGTGGGGTTGTTTA	CCTTCTTTCRAAATAACCTAAAACAAC
	RASSF1_M9	GGTYGGTTTTAGTTATAGTTGGATAATG	TAAACAACCCCACCCTCCTAAC
PIK3R1	PIK3R1_M2	GTTTGGGGTTGGTTGAAAGAT	CCTAACRAACCCTTCCTACCAC
	PIK3R1_M4	TGGAGYGGAGTTGGAGGAAGTAG	CACACCCRAAACTACTACTACCTACCTA
	PIK3R1_M5	GGAAAYGGGAGTTAGGATGG	CAACAACAACCCCRAATATATATACTC
	PIK3R1_M6	GTAGYGATTTTGGTTGTAGTTGGAG	CCATCCTAACTCCCRTTTCC
	PIK3R1_M7	TTTYGTGGTTTTTTAGTTGTAGTTAGG	CCAACAACCTACCCAAACTTAAC
	PIK3R1_M8	GAAATTTAGTTGGTTTTTTAATGAGGA	ACCTCCCCCCAACCTATTC
ITGAV	ITGAV_M1	TTGAGAGGTAGGATGGGTGAG	TCTTCTCTCRAAACTCCTACTACCTCT
	ITGAV_M2	AGGTAGTAGGAGTTTYGAGAGAAGAAG	AAACTCAACCCTCTTACCTACCC
	ITGAV_M3	GGGGTAGGTAAGAGGGTTGAG	ACTCCTCCTCCTTCCAAATCTC
NFKB1	NFKB1_M2	GGGGTAGGAAGAGGAGGTTT	AACCRAACCAAACCAATCAAC
	NFKB1_M3	GTTGATTGGTTTGGTTYGGTT	CCCTACCRAACCCCCACT
	NFKB1_M4	GGGAGGAGGTTGATAGTAGTTGAG	CACTCCAACCTTCTCACCATC
TAP2	TAP2_M1	GGTGGTTTAYGTTTGTAATTATAGTATTTTG	CTCACTCTTATCRCCCAAACTAAAATAC
	TAP2_M2	GTTAAGGTTTTTATTTTGGGTTGG	TCTCCAATTACAAAACATTCTCCA
	TAP2_M3	GGAGTGGGTAGTTATTTGGGTTG	CCAACCCAAAATAAAAACCTTAAC
APC	APC_M2	GGGTTAGGGTTAGGTAGGTTGTG	CATTCTATCTCCAATAACACCCTAAC
BRCA1	BRCA1_M1	GGGAGGAATTTTGTAAAGAAGAGG	ACRAACTAAAAAACTCCTCCAACAC
	BRCA1_M2	GGGGAGGYGGTAATGTAAAGAT	ACCCCTCAACCCCAATATTTA
	BRCA1_M3	AGTGATGTTTTGGGGTATTGG	AAACTCCTAACCTCATAACCAACC
	BRCA1_M4	GAGGTTAGGAGTTTTAGATTAGTTTGATT	CCATCCTCTCATACATACCAACC
CCND1	CCND1_M3	TAGGGTTTGATTTTYGTTTGTAGG	AAAACCCCAAAAATTCAAACTC
CDH1	CDH1_M1	GGAATTGTAAAGTATTTGTGAGTTTG	CTCCTCAAAACCCRAACTTTCT
CDKN2A	CDKN2A-2	GGGATATGGAGGGGGAGAT	CTTCTTCCTCTTTCCTCTTCCC
	CDKN2A-4	AATAAAATAAGGGGAATAGGGGAG	CCATCTTCCCACCCTCAA
	CDKN2A-6	GTAGTTAAGGGGGTAGGAGTGG	ACTACTACCCTAAACRCTAACTCCTCAA
	CDKN2A-7	TTGAGGAGTTAGYGTTTAGGGTAGTAGT	TCAATAATACTACRAAAACCACATATCTAAATC
	CDKN2A_M8	GTTTTTTAGGTTGGAGTGTAATGG	TCTATAATCCCAACATTCTAAAAAACC
	CDKN2A_M9	TTAGAGGATTTGAGGGATAGGGT	AACCAATCAACCRAAAACTCC
	CDKN2A_M10	GGAGTTTTYGGTTGATTGGTT	CCCAAAAAACCTCCCCTTT
BNIP3	BNIP3-1	GGTAAYGTGGATTTTGAGGTTGT	CCATCCTCCCCTTCCRTAC
	BNIP3-3	GGTTGYGGGATGTGTTTTAGTT	CAAACCTCTACCCCTCRCCC
	BNIP3-6	GGTGGGTYGGAGTTGAGYGT	TACACCRCRAAAACCCCTTAC
	BNIP3-7	GTAAGGGGTTTTYGGGGTGTA	CCTCTAAAAAATACCTCCCAATCC
HTATIP2	HTATIP2-1	TTTGGGTGAGTTGAGTTTAGTAGG	AAACATCCCACCTTCCCTAA
	HTATIP2-2	TTAGGGAAGGTGGGATGTTT	ACTACTAACATCACTAAACATACCCCAC
PRDM16	PRDM16-1	GGTAGGGAATGGGGTTGTG	CTAAACCTTCTACCTTAAATCCTCC
	PRDM16-2	GGAGGATTTAAGGTAGAAGGTTTAG	ACTCCTAACCTTACCCTCCCTAC
	PRDM16-3	GTAGGGAGGGTAAGGTTAGGAGT	AATAACCCRAACCCAAAAACCT
	PRDM16-4	AGGTTTTTGGGTTYGGGTTATT	AACTAAACCACCTTCRAAAACCC
	PRDM16-5	GGGTTTTYGAAGGTGGTTTAGTT	CTCCRCCACTACCCAAAC
	PRDM16-7	GAGGGGAGAATGTAGGAGAAAAG	CTACTACTCCCRCCCCAACC
	PRDM16-8	GGTTGGGGYGGGAGTAGTAG	CACTTATCTCTCCCCCCTCTC
	PRDM16-9	GAGAGGGGGGAGAGATAAGTG	CACTATCTTCATCTCCCCAACA
PTEN	PTEN-1	TTAGGGAGGGGGTTTGAGT	CTCCTCAACAACCAAAAACCTAA
PTX3	PTX3-1	GTAGGTTTGGGYGGGTTGTT	TCCAAAACACTAATCAACCTAACCT
	PTX3-2	AGGTTAGGTTGATTAGTGTTTTGGA	CTCCTTACCTACCRACAACCAA
SOCS1	SOCS1-1	GGGGAGGGTATTTATATGGTTTTA	ACTAAAAACCCCRCTACRCCAAC
	SOCS1-2	GTTGGYGTAGYGGGGTTTTTAGT	CCTACRAATTCTACTAAAAACCCCTAA
	SOCS1-3	TTAGGGGTTTTTAGTAGAATTYGTAGG	CAATCTCCACAACAACAAAACC

### Bisulfite conversion and multiplex amplification

Genomic DNA (about 400 ng) was subjected to sodium bisulfite modification using EZ DNA Methylation™-GOLD Kit (Zymo Research, Orange, CA, USA) according to the manufacturer's protocols. An un-methylated cytosine was converted to uracil when treated with bisulfite, whereas a methylated cytosine remained as cytosine [38]. A multiplex PCR was performed using the optimized primer sets. A 20 μl PCR reaction mixture was prepared for each reaction, including 1x buffer (TaKaRa, Tokyo, Japan), 3 mM Mg^2+^, 0.2 mM dNTP, 0.1 μM of each primer, 1U HotStarTaq polymerase (TaKaRa, Tokyo, Japan) and 2 μl of template DNA. The cycling program was 95°C for 2 min; 11 cycles of 94°C for 20 sec, 63°C for 40 sec with a decreasing temperature step of 0.5°C per cycle, 72°C for 1 min; then followed by 24 cycles of 94°C for 20 sec, 65°C for 30 sec, 72°C for 1 min; 72°C for 2 min.

### Index PCR and sequencing

PCR amplicons were then diluted and amplified using the indexed primers. Specifically, a 20 μl mixture was prepared for each reaction, including 1x buffer (NEB, MA, USA), 0.3 mM dNTP, 0.3 μM forward primer, 0.3 μM index primer, 1 U Q5^TM^ DNA polymerase (NEB, MA, USA) and 1 μL of diluted template (PCR amplicons from the previous step). The cycling program was 98°C for 30 sec; 11 cycles of 98°C for 10 sec, 65°C for 30 sec, 72°C for 30 sec; 72°C for 5 min. The PCR products (170 bp −270 bp) were separated by agarose electrophoresis and purified using the QIAquick Gel Extraction kit (Qiagen, Hilden, Germany). Libraries from different samples were quantified and pooled together, followed by sequencing on the Illumina MiSeq platform according to the manufacturer's protocols. Sequencing was performed with a 2 × 300 bp paired-end mode. Quality control of sequencing-reads was performed by FastQC (http://www.bioinformatics.bbsrc.ac.uk/projects/fastqc/). Filtered-reads were aligned back to the reference genome using the Bismark software (http://www.bioinformatics.babraham.ac.uk/projects/bismark/). After reads recalibration withUSEARCH [39], the methylation and haplotype were analyzed using the Perl script.

### Immunohistochemistry

Sections (4 μm) of formalin fixed, paraffin embedded tissues were prepared. The slides were dried at 56°C for 1 hour, then deparaffinized with fresh xylene and rehydrated through ethanol washes. Antigen retrieval was performed by citrate buffer incubation (pH 6.0) using a microwave oven for 10 min at 100°C. Slides were incubated for 15 min with 3% hydrogen peroxide, washed in PBS, and incubated with an appropriate primary antibody followed by a secondary antibody. Sections were counterstained, and examined by fluorescence microscopy. Antibodies and dilutions used in immunocytochemistry were as follows: rabbit anti-PTEN (1:100); rabbit anti-RASSF1 (1:100); rabbit anti- PIK3R1 (1:100); rabbit anti IgG (1:400). The integrated optical density (IOD) was calculated for each sample [40]. For semi-quantitative analysis of the degree of staining, slides were independently scored by two pathologists. The scores were defined as follows: 0 (< 5% positive tumor cells); 1 (≤ 25% positive tumor cells); 2 (26–50% positive tumor cells); 3 (51–75% positive tumor cells); and 4 (> 75% positive tumor cells). Staining intensity was graded as: 0 (no staining); 1 (weak staining: light yellow); 2 (moderate staining: yellow brown); and 3 (strong staining: brown). Staining index (SI) was calculated as the product of staining intensity score and the proportion of positive tumor cells [41]. An SI score of 9–12 indicated strong positive (+++); 5–8 indicated positive (++); 1–4 indicated weakly positive (+); 0 indicated negative (−) staining.

### Statistical analysis

We used the IBM SPSS Statistics 19.0 (IBM Corp., NY, USA) and the R program (https://www.r-project.org/) to analyze the data. Individual and cumulative methylation statuses of candidate genes were analyzed. We used the *t-test*, ANOVA or nonparametric test to compare the differences of methylation between groups. Considering the false positive caused by multiple comparisons, the Bonferroni correction was applied. The receiver operative characteristics (ROC) curve was drafted to reflect the diagnostic value of biomarkers. The area under the curve (AUC) together with 95% confidence interval (CI) were calculated.

## SUPPLEMENTARY MATERIALS





## References

[1] Napier KJ, Scheerer M, Misra S (2014). Esophageal cancer: A Review of epidemiology, pathogenesis, staging workup and treatment modalities. World J Gastrointest Oncol.

[2] Arnold M, Soerjomataram I, Ferlay J, Forman D (2015). Global incidence of oesophageal cancer by histological subtype in 2012. Gut.

[3] Ingelfinger JR, Rustgi AK, El-Serag HB (2014). Esophageal Carcinoma. New England Journal of Medicine.

[4] Vizcaino AP, Moreno V, Lambert R, Parkin DM (2002). Time trends incidence of both major histologic types of esophageal carcinomas in selected countries, 1973–1995. Int J Cancer.

[5] Enzinger PC, Mayer RJ (2003). Esophageal cancer. N Engl J Med.

[6] Naidoo R, Ramburan A, Reddi A, Chetty R (2005). Aberrations in the mismatch repair genes and the clinical impact on oesophageal squamous carcinomas from a high incidence area in South Africa. J Clin Pathol.

[7] Cao H, Li E, Xu L (2014). Abstract 2205: A three-gene signature predicts esophageal squamous cell carcinoma prognosis. Cancer Res.

[8] Wang GQ (2001). [30-year experiences on early detection and treatment of esophageal cancer in high risk areas]. Zhongguo Yi Xue Ke Xue Yuan Xue Bao.

[9] Feinberg AP, Ohlsson R, Henikoff S (2006). The epigenetic progenitor origin of human cancer. Nat Rev Genet.

[10] Jia Y, Yang Y, Brock MV, Cao B, Zhan Q, Li Y, Yu Y, Herman JG, Guo M (2012). Methylation of TFPI-2 is an early event of esophageal carcinogenesis. Epigenomics.

[11] Soto J, Rodriguez-Antolin C, Vallespin E, de Castro Carpeno J, Ibanez de Caceres I (2016). The impact of next-generation sequencing on the DNA methylation-based translational cancer research. Transl Res.

[12] Siegfried Z, Simon I (2010). DNA methylation and gene expression. Wiley Interdiscip Rev Syst Biol Med.

[13] Gravina GL, Festuccia C, Marampon F, Popov VM, Pestell RG, Zani BM, Tombolini V (2010). Biological rationale for the use of DNA methyltransferase inhibitors as new strategy for modulation of tumor response to chemotherapy and radiation. Mol Cancer.

[14] Das PM, Singal R (2004). DNA methylation and cancer. J Clin Oncol.

[15] Sun B, Hu L, Luo ZY, Chen XP, Zhou HH, Zhang W (2016). DNA methylation perspectives in the pathogenesis of autoimmune diseases. Clin Immunol.

[16] Nestler EJ, Pena CJ, Kundakovic M, Mitchell A, Akbarian S (2016). Epigenetic Basis of Mental Illness. Neuroscientist.

[17] Zhong J, Agha G, Baccarelli AA (2016). The Role of DNA Methylation in Cardiovascular Risk and Disease: Methodological Aspects, Study Design, and Data Analysis for Epidemiological Studies. Circ Res.

[18] Baba Y, Watanabe M, Baba H (2013). Review of the alterations in DNA methylation in esophageal squamous cell carcinoma. Surg Today.

[19] Portela A, Esteller M (2010). Epigenetic modifications and human disease. Nat Biotechnol.

[20] Wang J, Sasco AJ, Fu C, Xue H, Guo G, Hua Z, Zhou Q, Jiang Q, Xu B (2008). Aberrant DNA methylation of P16, MGMT, and hMLH1 genes in combination with MTHFR C677T genetic polymorphism in esophageal squamous cell carcinoma. Cancer epidemiology, biomarkers & prevention.

[21] Ma K, Cao B, Guo M (2016). The detective, prognostic, and predictive value of DNA methylation in human esophageal squamous cell carcinoma. Clin Epigenetics.

[22] Takahashi T, Matsuda Y, Yamashita S, Hattori N, Kushima R, Lee YC, Igaki H, Tachimori Y, Nagino M, Ushijima T (2013). Estimation of the fraction of cancer cells in a tumor DNA sample using DNA methylation. PloS one.

[23] Ashktorab H, Daremipouran M, Goel A, Varma S, Leavitt R, Sun X, Brim H (2014). DNA methylome profiling identifies novel methylated genes in African American patients with colorectal neoplasia. Epigenetics.

[24] Reis-Filho JS (2009). Next-generation sequencing. Breast Cancer Res.

[25] Sonnet M, Claus R, Becker N, Zucknick M, Petersen J, Lipka DB, Oakes CC, Andrulis M, Lier A, Milsom MD, Witte T, Gu L, Kim-Wanner SZ (2014). Early aberrant DNA methylation events in a mouse model of acute myeloid leukemia. Genome Med.

[26] Duhoux FP, Ameye G, Montano-Almendras CP, Bahloula K, Mozziconacci MJ, Laibe S, Wlodarska I, Michaux L, Talmant P, Richebourg S, Lippert E, Speleman F, Herens C (2012). PRDM16 (1p36) translocations define a distinct entity of myeloid malignancies with poor prognosis but may also occur in lymphoid malignancies. Br J Haematol.

[27] Tan SX, Hu RC, Liu JJ, Tan YL, Liu WE (2014). Methylation of PRDM2, PRDM5 and PRDM16 genes in lung cancer cells. Int J Clin Exp Pathol.

[28] Cizkova M, Vacher S, Meseure D, Trassard M, Susini A, Mlcuchova D, Callens C, Rouleau E, Spyratos F, Lidereau R, Bieche I (2013). PIK3R1 underexpression is an independent prognostic marker in breast cancer. BMC Cancer.

[29] Sen S, Bhattacharjee S, Mukhopadhyay I, Mandal P, Sharma S, Roy Chowdhury R, Sengupta S (2015). Abstract 1056: Impact of host methylome on cervical cancer pathogenesis. Cancer Res.

[30] Kaz AM, Grady WM (2014). Epigenetic biomarkers in esophageal cancer. Cancer Lett.

[31] Kishino T, Niwa T, Yamashita S, Takahashi T, Nakazato H, Nakajima T, Igaki H, Tachimori Y, Suzuki Y, Ushijima T (2016). Integrated analysis of DNA methylation and mutations in esophageal squamous cell carcinoma. Mol Carcinog.

[32] Byun DS, Lee MG, Chae KS, Ryu BG, Chi SG (2001). Frequent epigenetic inactivation of RASSF1A by aberrant promoter hypermethylation in human gastric adenocarcinoma. Cancer Res.

[33] Lo KW, Kwong J, Hui AB, Chan SY, To KF, Chan AS, Chow LS, Teo PM, Johnson PJ, Huang DP (2001). High frequency of promoter hypermethylation of RASSF1A in nasopharyngeal carcinoma. Cancer Res.

[34] Zhang Y, Jeltsch A (2010). The application of next generation sequencing in DNA methylation analysis. Genes.

[35] Sham PC, Purcell SM (2014). Statistical power and significance testing in large-scale genetic studies. Nat Rev Genet.

[36] Jahr S, Hentze H, Englisch S, Hardt D, Fackelmayer FO, Hesch RD, Knippers R (2001). DNA fragments in the blood plasma of cancer patients: quantitations and evidence for their origin from apoptotic and necrotic cells. Cancer Res.

[37] Wang JM, Xu B, Hsieh CC, Jiang QW (2005). Longitudinal trends of stomach cancer and esophageal cancer in Yangzhong County: a high-incidence rural area of China. European journal of gastroenterology & hepatology.

[38] Guo S, Yan F, Xu J, Bao Y, Zhu J, Wang X, Wu J, Li Y, Pu W, Liu Y, Jiang Z, Ma Y, Chen X (2015). Identification and validation of the methylation biomarkers of non-small cell lung cancer (NSCLC). Clin Epigenetics.

[39] Edgar RC (2010). Search and clustering orders of magnitude faster than BLAST. Bioinformatics.

[40] Wu D, Tao J, Xu B, Li P, Lu Q, Zhang W (2012). Downregulation of Dicer, a component of the microRNA machinery, in bladder cancer. Mol Med Rep.

[41] Su P, Zhang Q, Yang Q (2010). Immunohistochemical analysis of Metadherin in proliferative and cancerous breast tissue. Diagn Pathol.

