# Supplementary material for: Accumulated promoter methylation as a potential biomarker for esophageal cancer
Source: Oncotarget. 2016 Nov 22;8(1):679–91. doi: 10.18632/oncotarget.13510 (PMC5352188; doi:10.18632/oncotarget.13510)
Supplement: Supplementary file 1 [file oncotarget-08-679-s001.pdf]

Accumulated promoter methylation as a potential biomarker for esophageal cancer

Supplementary Materials

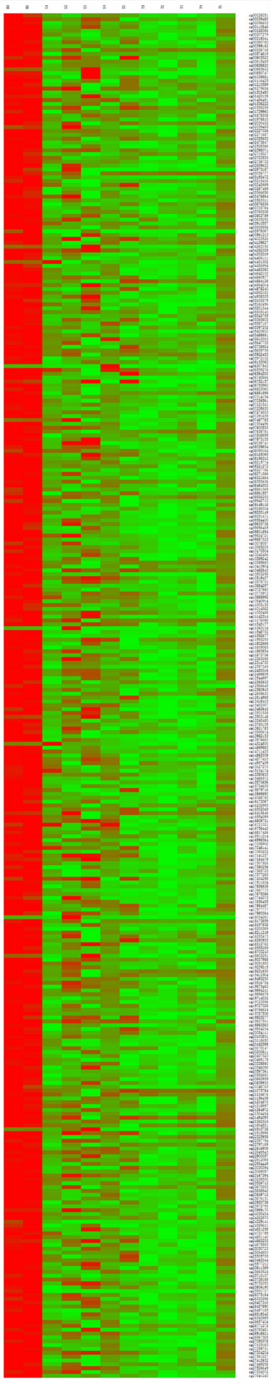

Supplementary Figure S1: Hierarchical clustering of methylation between esophageal cancer tissues and normal controls. The heat map of these genes shows different methylation between cancer, remote normal-appearing and healthy control tissues. Green indicates hypermethylation, while red indicates hypomethylation. Columns represent samples. Rows represent CG loci.

Supplementary Figure S2: Description of candidate genes. See Supplementary_Figure_S2
