# Supplementary material for: Accumulated promoter methylation as a potential biomarker for esophageal cancer
Source: Oncotarget. 2016 Nov 22;8(1):679–91. doi: 10.18632/oncotarget.13510 (PMC5352188; doi:10.18632/oncotarget.13510)
Supplement: Supplementary file 2 [file oncotarget-08-679-s002.docx]

**Supplement figure 2. Description of candidate genes**

| Genes | Description |
| --- | --- |
| APC | This gene encodes a tumor suppressor protein that acts as an antagonist of the Wnt signaling pathway. It is also involved in other processes including cell migration and adhesion, transcriptional activation, and apoptosis. Defects in this gene cause familial adenomatous polyposis (FAP), an autosomal dominant pre-malignant disease that usually progresses to malignancy. Disease-associated mutations tend to be clustered in a small region designated the mutation cluster region (MCR) and result in a truncated protein product. |
| BRCA1 | This gene encodes a nuclear phosphoprotein that plays a role in maintaining genomic stability, and it also acts as a tumor suppressor. The encoded protein combines with other tumor suppressors, DNA damage sensors, and signal transducers to form a large multi-subunit protein complex known as the BRCA1-associated genome surveillance complex (BASC). This gene product associates with RNA polymerase II, and through the C-terminal domain, also interacts with histone deacetylase complexes. This protein thus plays a role in transcription, DNA repair of double-stranded breaks, and recombination. Mutations in this gene are responsible for approximately 40% of inherited breast cancers and more than 80% of inherited breast and ovarian cancers. Alternative splicing plays a role in modulating the subcellular localization and physiological function of this gene. Many alternatively spliced transcript variants, some of which are disease-associated mutations, have been described for this gene, but the full-length natures of only some of these variants has been described. A related pseudogene, which is also located on chromosome 17, has been identified. |
| CCND1 | The protein encoded by this gene belongs to the highly conserved cyclin family, whose members are characterized by a dramatic periodicity in protein abundance throughout the cell cycle. Cyclins function as regulators of CDK kinases. Different cyclins exhibit distinct expression and degradation patterns which contribute to the temporal coordination of each mitotic event. This cyclin forms a complex with and functions as a regulatory subunit of CDK4 or CDK6, whose activity is required for cell cycle G1/S transition. This protein has been shown to interact with tumor suppressor protein Rb and the expression of this gene is regulated positively by Rb. Mutations, amplification and overexpression of this gene, which alters cell cycle progression, are observed frequently in a variety of tumors and may contribute to tumorigenesis. |
| CDH1 | This gene encodes a classical cadherin of the cadherin superfamily. Alternative splicing results in multiple transcript variants, at least one of which encodes a preproprotein that is proteolytically processed to generate the mature glycoprotein. This calcium-dependent cell-cell adhesion protein is comprised of five extracellular cadherin repeats, a transmembrane region and a highly conserved cytoplasmic tail. Mutations in this gene are correlated with gastric, breast, colorectal, thyroid and ovarian cancer. Loss of function of this gene is thought to contribute to cancer progression by increasing proliferation, invasion, and/or metastasis. The ectodomain of this protein mediates bacterial adhesion to mammalian cells and the cytoplasmic domain is required for internalization. This gene is present in a gene cluster with other members of the cadherin family on chromosome 16. |
| CDKN2A | This gene generates several transcript variants which differ in their first exons. At least three alternatively spliced variants encoding distinct proteins have been reported, two of which encode structurally related isoforms known to function as inhibitors of CDK4 kinase. The remaining transcript includes an alternate first exon located 20 Kb upstream of the remainder of the gene; this transcript contains an alternate open reading frame (ARF) that specifies a protein which is structurally unrelated to the products of the other variants. This ARF product functions as a stabilizer of the tumor suppressor protein p53 as it can interact with, and sequester, the E3 ubiquitin-protein ligase MDM2, a protein responsible for the degradation of p53. In spite of the structural and functional differences, the CDK inhibitor isoforms and the ARF product encoded by this gene, through the regulatory roles of CDK4 and p53 in cell cycle G1 progression, share a common functionality in cell cycle G1 control. This gene is frequently mutated or deleted in a wide variety of tumors, and is known to be an important tumor suppressor gene. |
| BNIP3 | This gene is encodes a mitochondrial protein that contains a BH3 domain and acts as a pro-apoptotic factor. The encoded protein interacts with anti-apoptotic proteins, including the E1B 19 kDa protein and Bcl2. This gene is silenced in tumors by DNA methylation. |
| HTATIP2 | HTATIP2, also known as 30-kilodalton HIV-1 Tat interacting protein (TIP30) or CC3, typically functions as a tumor suppressor and was initially identified in the highly metastatic human variant small cell lung carcinoma (SCLC) in comparison with the less metastatic classic SCLC cell lines. HTATIP2 frequently exhibits downregulation in various tumor cells, such as breast cancer, colon cancer, pancreatic cancer, melanoma, glioblastoma, neuroblastoma, SCLC, and HCC cells. |
| PRDM16 | The reciprocal translocation t(1;3)(p36;q21) occurs in a subset of myelodysplastic syndrome (MDS) and acute myeloid leukemia (AML). This gene is located near the 1p36.3 breakpoint and has been shown to be specifically expressed in the t(1:3)(p36,q21)-positive MDS/AML. The protein encoded by this gene is a zinc finger transcription factor and contains an N-terminal PR domain. The translocation results in the overexpression of a truncated version of this protein that lacks the PR domain, which may play an important role in the pathogenesis of MDS and AML. Alternatively spliced transcript variants encoding distinct isoforms have been reported. |
| PTEN | This gene was identified as a tumor suppressor that is mutated in a large number of cancers at high frequency. The protein encoded by this gene is a phosphatidylinositol-3,4,5-trisphosphate 3-phosphatase. It contains a tensin like domain as well as a catalytic domain similar to that of the dual specificity protein tyrosine phosphatases. Unlike most of the protein tyrosine phosphatases, this protein preferentially dephosphorylates phosphoinositide substrates. It negatively regulates intracellular levels of phosphatidylinositol-3,4,5-trisphosphate in cells and functions as a tumor suppressor by negatively regulating AKT/PKB signaling pathway. The use of a non-canonical (CUG) upstream initiation site produces a longer isoform that initiates translation with a leucine, and is thought to be preferentially associated with the mitochondrial inner membrane. This longer isoform may help regulate energy metabolism in the mitochondria. A pseudogene of this gene is found on chromosome 9. Alternative splicing and the use of multiple translation start codons results in multiple transcript variants encoding different isoforms. |
| PTX3 | This gene encodes a member of the pentraxin protein family. The expression of this protein is induced by inflammatory cytokines in response to inflammatory stimuli in several mesenchymal and epithelial cell types, particularly endothelial cells and mononuclear phagocytes. The protein promotes fibrocyte differentiation and is involved in regulating inflammation and complement activation. It also plays a role in angiogenesis and tissue remodeling. The protein serves as a biomarker for several inflammatory conditions. |
| SOCS1 | This gene encodes a member of the STAT-induced STAT inhibitor (SSI), also known as suppressor of cytokine signaling (SOCS), family. SSI family members are cytokine-inducible negative regulators of cytokine signaling. The expression of this gene can be induced by a subset of cytokines, including IL2, IL3 erythropoietin (EPO), CSF2/GM-CSF, and interferon (IFN)-gamma. The protein encoded by this gene functions downstream of cytokine receptors, and takes part in a negative feedback loop to attenuate cytokine signaling. Knockout studies in mice suggested the role of this gene as a modulator of IFN-gamma action, which is required for normal postnatal growth and survival. |
| RASSF1 | This gene encodes a protein similar to the RAS effector proteins. Loss or altered expression of this gene has been associated with the pathogenesis of a variety of cancers, which suggests the tumor suppressor function of this gene. The inactivation of this gene was found to be correlated with the hypermethylation of its CpG-island promoter region. The encoded protein was found to interact with DNA repair protein XPA. The protein was also shown to inhibit the accumulation of cyclin D1, and thus induce cell cycle arrest. Several alternatively spliced transcript variants of this gene encoding distinct isoforms have been reported. |
| PIK3R1 | Phosphatidylinositol 3-kinase phosphorylates the inositol ring of phosphatidylinositol at the 3-prime position. The enzyme comprises a 110 kD catalytic subunit and a regulatory subunit of either 85, 55, or 50 kD. This gene encodes the 85 kD regulatory subunit. Phosphatidylinositol 3-kinase plays an important role in the metabolic actions of insulin, and a mutation in this gene has been associated with insulin resistance. Alternative splicing of this gene results in four transcript variants encoding different isoforms. |
| ITGAV | The product of this gene belongs to the integrin alpha chain family. Integrins are heterodimeric integral membrane proteins composed of an alpha subunit and a beta subunit that function in cell surface adhesion and signaling. The encoded preproprotein is proteolytically processed to generate light and heavy chains that comprise the alpha V subunit. This subunit associates with beta 1, beta 3, beta 5, beta 6 and beta 8 subunits. The heterodimer consisting of alpha V and beta 3 subunits is also known as the vitronectin receptor. This integrin may regulate angiogenesis and cancer progression. Alternative splicing results in multiple transcript variants. Note that the integrin alpha 5 and integrin alpha V subunits are encoded by distinct genes. |
| NFKB1 | This gene encodes a 105 kD protein which can undergo cotranslational processing by the 26S proteasome to produce a 50 kD protein. The 105 kD protein is a Rel protein-specific transcription inhibitor and the 50 kD protein is a DNA binding subunit of the NF-kappa-B (NFKB) protein complex. NFKB is a transcription regulator that is activated by various intra- and extra-cellular stimuli such as cytokines, oxidant-free radicals, ultraviolet irradiation, and bacterial or viral products. Activated NFKB translocates into the nucleus and stimulates the expression of genes involved in a wide variety of biological functions. Inappropriate activation of NFKB has been associated with a number of inflammatory diseases while persistent inhibition of NFKB leads to inappropriate immune cell development or delayed cell growth. Alternative splicing results in multiple transcript variants encoding different isoforms, at least one of which is proteolytically processed. |
| TAP2 | The membrane-associated protein encoded by this gene is a member of the superfamily of ATP-binding cassette (ABC) transporters. ABC proteins transport various molecules across extra- and intra-cellular membranes. ABC genes are divided into seven distinct subfamilies (ABC1, MDR/TAP, MRP, ALD, OABP, GCN20, White). This protein is a member of the MDR/TAP subfamily. Members of the MDR/TAP subfamily are involved in multidrug resistance. This gene is located 7 kb telomeric to gene family member ABCB2. The protein encoded by this gene is involved in antigen presentation. This protein forms a heterodimer with ABCB2 in order to transport peptides from the cytoplasm to the endoplasmic reticulum. Mutations in this gene may be associated with ankylosing spondylitis, insulin-dependent diabetes mellitus, and celiac disease. Alternative splicing of this gene produces products which differ in peptide selectivity and level of restoration of surface expression of MHC class I molecules. |
